# Shifts in the Composition of the Microbiota of Stored Wheat Grains in Response to Fumigation

**DOI:** 10.3389/fmicb.2019.01098

**Published:** 2019-05-17

**Authors:** Manoj Kumar Solanki, Ahmed Abdelfattah, Malka Britzi, Varda Zakin, Michael Wisniewski, Samir Droby, Edward Sionov

**Affiliations:** ^1^Institute of Postharvest and Food Sciences, Agricultural Research Organization – The Volcani Center, Rishon LeZion, Israel; ^2^Dipartimento di Agraria, Università Mediterranea di Reggio Calabria, Calabria, Italy; ^3^Department of Ecology, Environment and Plant Sciences, Stockholm University, Stockholm, Sweden; ^4^National Residue Control Laboratory, Kimron Veterinary Institute, Beit Dagan, Israel; ^5^United States Department of Agriculture, Agricultural Research Service, Kearneysville, WV, United States

**Keywords:** stored wheat grain, microbiome analysis, phosphine fumigation, mycotoxigenic fungi, mycotoxins

## Abstract

While the wheat-associated microbiome is of major agricultural importance, little is known about the alterations in wheat grain microbial community composition during storage. Characterization of the bacterial and fungal communities in stored wheat grains revealed the impact of phosphine fumigation, one of the most effective methods to eliminate insects in stored commodities, on the composition of the wheat grain microbiome. High-throughput amplicon sequencing of the bacterial 16S rRNA gene and fungal internal transcribed spacer (ITS) region was used to analyze the wheat grain microbiome at different times over as 6 months period of storage. Higher bacterial diversity was found across the samples during the first (immediately after harvest) and second (3 months later) time points, with a predominance of *Proteobacteria*, *Firmicutes*, *Actinobacteria*, *Bacteroidetes* and *Planctomycetes*. A two-fold decrease in the number of bacterial operational taxonomic units (OTUs) was observed in wheat grains at the last time point (6 months later), following phosphine treatment. In contrast to the effect of phosphine on bacteria, it did not affect fungal diversity in stored grains. The majority of fungal sequences were assigned to *Ascomycota*, followed by *Basidiomycota*, *Glomeromycota*, and unidentified fungi, which were evenly distributed throughout the storage period. Alpha and beta diversity analyses were confirmed by examination of the cultured microbial taxa obtained from the stored wheat grains. Mycotoxin analysis of wheat grains collected after phosphine fumigation revealed the presence of *Fusarium* toxins, primarily deoxynivalenol (DON). Several mycotoxigenic *Fusarium* spp. were also detected in the same samples. Results of the present study indicate that microbiome of stored, whole wheat grains was strongly affected by phosphine fumigation, which changed the structure of the microbial community leading to shifts in species composition toward mycotoxigenic strains. A better understanding of the complex interactions within the microbial communities of stored grains will assist in the development of novel biocontrol strategies to overcome mycotoxin contamination.

## Introduction

Wheat is one of the most important cultivated cereals produced and consumed globally. It provides, on average, about 20% of the daily caloric requirement and about 21% of the daily protein intake in the human diet (Shiferaw et al., 2013). The availability of high-quality wheat grains is essential for maintaining the stability of the world’s food supply and for global food security. Wheat grains are colonized by complex microbial communities that play different roles in grain quality and susceptibility to disease (Links et al., 2014). Some of the bacteria and fungi that are associated with seeds can be harmful to human health and cause plant diseases, while others can have beneficial effects on the host and actually improve nutrient uptake and increase tolerance to biotic and abiotic stresses through multiple mechanisms (Brader et al., 2017). For example, specific species of *Fusarium*, *Aspergillus*, *Penicillium*, and *Alternaria* can cause spoilage in stored wheat grains and produce mycotoxins that severely decrease crop value and are harmful to animal and human health (Placinta et al., 1999; Magan et al., 2010). Conversely, the plant protective ability of some bacterial epiphytes and endophytes against fungal pathogens has been reported in several crops (Links et al., 2014; Mousa et al., 2016; Gdanetz and Trail, 2017). A better knowledge of the composition and dynamics of wheat-grain-associated microbiota is needed to identify novel beneficial microorganisms that may improve crop health and suppress the growth of potential pathogens in a sustainable manner. Several studies on the composition of the rhizosphere microbiome of wheat and other parts of the wheat plant have been conducted (Ofek et al., 2014; Hartmann et al., 2015; Gdanetz and Trail, 2017; Mahoney et al., 2017). Few studies, however, have characterized the microbiota of wheat grains and the seeds of other crop species (Links et al., 2014; Yuan et al., 2018).

In addition to fungal pathogens that cause seed deterioration during storage, insects can infest and spoil grain that is handled or stored improperly. Thus, a fumigation treatment is often required to prevent insect infestations in stored grains. Phosphine gas is by far the most common fumigation agent used worldwide for the treatment of stored grain against insects, and has recently increased in usage following the prohibition of methyl bromide, an alternative fumigant. Although phosphine is very toxic to insects, it does not leave toxic residues after treatment or impact seed viability (Bell, 2000). While this fumigant is known to have broad biological activity (Berners-Price and Sadler, 1988; Nath et al., 2011), its effect on the microbial community of stored wheat grains is largely unknown. Few studies have been reported on the effect of phosphine on the growth of molds in stored wheat and corn grains (Hocking and Banks, 1991; Castro et al., 2000). These studies reported that phosphine only causes a slight decrease in the population of *Aspergillus flavus* in stored maize and *Eurotium chevalieri*, *A. flavus*, or *Aspergillus parasiticus* in stored wheat grains.

The objective of the present study was to characterize the composition of the fungal and bacterial communities in stored wheat grains, which produced annually for human and animal consumption, and to determine the impact of phosphine fumigation on the wheat grain microbiome during storage. Amplicon-based high-throughput sequencing (HTS) and chemical analysis of mycotoxins were used to characterize the wheat grain microbiome at different time points over a storage period of 6 months. Significant changes in the composition of the microbial community were identified when phosphine treatment was applied to stored wheat grains. *Fusarium*-related toxins were also detected in stored wheat grains as a result of shifts in the microbial population profiles which took place following fumigation with phosphine.

## Materials and Methods

### Wheat Grain Samples

Wheat grain (*Triticum aestivum*) samples were collected from seven wheat grain storage facilities located in northern and southern districts of Israel. Samples were designated as T1 when obtained immediately after harvest during the first week of storage (June 2016), T2 after storage for 3 months, and T3 after storage for 6 months. Four months after harvest (before collecting T3 samples) the stored grains in all locations were exposed to phosphine (3 g/m^3^) for a period of 10 days to control insect pests. In each storage facility, three samples (1 kg of grain each), destined for human consumption, were collected at each time point (3 samples × 7 storage sites = a total of 21 samples at each time point) in equal proportions from the front face and the center, at points located 1 m in horizontal depth within the grain mass, and from areas close to the walls. Grain temperature and moisture content were in the ranges of 27–33°C and 10.5–12.9%, respectively, throughout the study. The collected samples were kept in sterile plastic bags during transport to the laboratory and on the same day a 100 g aliquot was taken from each sample, thoroughly mixed, frozen in liquid nitrogen, freeze dried and milled into a fine powder with a grain grinder. The grain grinder was cleaned and disinfected with 70% ethanol solution between sample grinding. Powders were stored at 4°C until further pending analysis (2–4 weeks).

### DNA Extraction, Amplification and Sequencing

DNA extraction from wheat grain samples was performed as previously described (Sadhasivam et al., 2017). Total DNA was extracted from 300 mg of wheat powder using a lysis buffer containing hexadecyltrimethylammonium bromide (CTAB). Ten milliliters of DNA extraction buffer (l.0 M Tris–HCl, pH 7.5; 1% (*w*/*v*) CTAB; 5 M NaCl; 0.5 M EDTA; 1% (*v*/*v*) 2-mercaptoethanol; and proteinase K at 0.3 mg/ml) were added to the powder, mixed gently, and the mixture was incubated at 65°C for 30 min. The extracts were cooled prior to the addition of an equal volume of chloroform, gently mixed and centrifuged at 6000 rpm for 10 min. The aqueous supernatant was recovered and an equal volume of 2-propanol was added. The DNA was precipitated by centrifugation at 4800 ×*g* for 5 min and resuspended in TE buffer solution (Tris-EDTA, pH 8.0) containing RNase A at 10 μg/ml. DNA was further purified by phenol-chloroform extraction. Finally, the DNA was precipitated with 100% ethanol containing 3 M sodium acetate, rinsed in 70% (*v*/*v*) ethanol and resuspended in TE buffer. The purity of the extracted DNA was assayed with a NanoDrop One spectrophotometer (Thermo Fisher Scientific, Wilmington, DE, United States), and the total DNA concentration in each sample was adjusted to 50 ng/μl. The universal primers 515F/926R and 5F/86R were used to amplify the 16S and ITS2 rRNA gene regions of bacteria and fungi, respectively (Supplementary Table S1). The primers were modified to include Illumina adapters^1^ for subsequent multiplexing. PCR amplification of each sample was performed in triplicate. The PCR mixture (25 μl) contained 12.5 μl 2× DreamTaq green PCR master-mix (Thermo Fisher Scientific, Lithuania), 1 μl of each primer (5 μM), and 1 μl of DNA template. Nuclease-free water (Thermo Fisher Scientific, Lithuania) replaced template DNA in negative controls. All amplicons and amplification mixtures including negative controls were sequenced using Illumina MiSeq V3 (2 × 300 bp) chemistry.

### Data Analysis

Illumina adaptors were clipped and low-quality reads removed by Trimmomatic 0.36 (Bolger et al., 2014) using a sliding window trimming, cutting once the average quality within the window of 4 bases falls below a quality threshold of 15. Paired-end reads were merged utilizing PEAR (Zhang et al., 2014) for 16S rRNA gene, and PANDAseq (Masella et al., 2012) for ITS rRNA gene region sequences with default parameters. Chimeric sequences were identified and removed using USEARCH (Edgar, 2010; McDonald et al., 2012) for 16S rRNA gene, and VSEARCH 1.4.0 (Rognes et al., 2016) for ITS rRNA gene region sequences. UCLUST algorithm (Edgar, 2010), as implemented in QIIME 1.9.1 (Caporaso et al., 2010) was used to cluster sequences queried against the Greengenes 13_8_97 database for 16S rRNA gene (DeSantis et al., 2006), and for ITS UNITE dynamic database released on 01.12.2017 (Abarenkov et al., 2010) at a similarity threshold of 97%, respectively. Sequences that failed to cluster against the database were *de novo* clustered using the same algorithm. After removing singletons, the most abundant sequences in each OTU were selected as representative sequences and used for the taxonomic assignment using the BLAST algorithm (Altschul et al., 1990; McDonald et al., 2012) as implemented in QIIME 1.9.1. The OTU table was normalized by rarefaction to an even sequencing depth in order to remove sample heterogeneity. The rarefied OTU table was used to calculate alpha diversity indices including Observed Species (Sobs), and Shannon metrics. Alpha diversities were compared based on a two-sample *t*-test using non-parametric (Monte Carlo) methods and 999 Monte Carlo permutations. Results were visualized in boxplots figures.

Metagenome Seq’s Cumulative Sum Scaling (CSS) (Paulson et al., 2013) was used as a normalization method for other downstream analyses. The CSS normalized OTUs table was analyzed using Bray Curtis metrics (Bray and Curtis, 1957) and utilized to evaluate β-diversity and construct PCoA plots using Emperor (Lozupone and Knight, 2005). Similarity in community composition was tested via ANOSIM in QIIME 1.9.1 using 999 permutations. Differential OTU abundance of the most abundant taxa (≥0.1%) between sample groups were determined using a *t*-test and the Kruskal-Wallis test (Kruskal and Wallis, 1952). In all tests, significance was determined using 999 Monte Carlo permutations, and the false discovery rate (FDR) was used to adjust the calculated *P*-values and when the FDR *P* < 0.05 it was considered significant.

### Microbial Isolation and Identification

Grains samples (10 g each) were shaken on an orbital shaker at 150 rpm in 90 ml sterile saline (0.9% NaCl) solution at room temperature for 1 h, then 100 μL of serial 1/10 dilutions were plated on Luria-Bertani (LB) agar plates for isolation of bacteria and Potato Dextrose Agar (PDA) plates supplemented with chloramphenicol (20 μg/ml) for isolation of filamentous fungi and yeasts. LB agar plates were incubated at 37°C for 24–48 h, whereas PDA plates were incubated at 28°C for 48–72 h. Bacterial colonies were randomly selected from the plates and streaked on fresh culture media to obtain pure cultures. Filamentous fungi and yeasts were transferred singly to PDA plates and subcultured twice to obtain a pure culture.

DNA was extracted from each bacterial strain using a lysozyme lysis method described by De et al. (2010). Fungal DNA extraction was performed on lyophilized mycelium/yeasts cells using CTAB-based method as previously described (Sadhasivam et al., 2017). DNA quality and yield were determined using a NanoDrop One spectrophotometer. The 16S rRNA gene in bacteria and ITS rRNA gene region in fungi were amplified with universal primers 27F/1492R and ITS1/ITS4, respectively (Supplementary Table S1). PCR products were purified and sequenced through standard Sanger sequencing; sequences were identified via BLAST matches to the NCBI database^2^ and deposited as accession number MK229027–MK229144 (Bacterial 16S rRNA gene) and MK226203–MK226306 (Fungal ITS rRNA gene region).

### Quantitative Real-Time PCR

qPCR was performed to quantify the presence of mycotoxigenic fungi, such as *Fusarium, Aspergillus*, and *Alternaria* spp., in the stored grains at different time points. A broad-spectrum primer pair ITSPF/ITSPR was used to target the fungal ITS region and single genus-specific TaqMan probes were tested for their ability to detect fungal DNA (Supplementary Table S2). The probes were labeled on the 5′ end with the fluorescent reporters FAM, HEX and TEX, and on the 3′ end with TAMRA, BHQ1, and BHQ2. The qPCR reactions were carried out using qPCRBIO Probe Mix (PCR Biosystems, London, United Kingdom), 10 μM of each primer, 10 μM of each probe, and 5 ng of genomic DNA template. Amplification reactions were performed in a total reaction volume of 10 μl and were run on the Eco Real-Time PCR System (Illumina) with the following program: 2 min at 50°C [Uracil-DNA glycosylases (UDG) activation], 2 min at 95°C (denaturation and Taq polymerase activation), an amplification program of 45 cycles at 95°C for 15 s, 55°C for 15 s, and 60°C for 15 s. Real-time qPCR reactions were performed in triplicate for each biological replicate and each sampling date contained three biological replicates. The cycle threshold value (Ct), which refers to the cycle number where the sample’s fluorescence significantly increases above the background level, was calculated automatically by the Eco software. The detection limit of the probes was assessed under optimized PCR conditions with the DNA extracted from pure cultures of *Fusarium proliferatum* (NRRL 31866), *A. flavus* (NRRL 3518), and *Alternaria infectoria* (F11, isolated from stored wheat grains). Serial dilutions were made of DNA from the three fungal species. The real-time PCR data indicated that the Ct values correlated well with known DNA quantities from 8 pg to 5 ng, with *R*^2^ values of 0.98 (slope -1.70) for *Fusarium*, 0.99 (slope -1.48) for *Aspergillus*, and 0.99 (slope -1.72) for *Alternaria* (Supplementary Figure S1).

To test the efficacy of the method, sterilized wheat grain samples (10 g) were inoculated with each of *A. flavus*, *F. proliferatum*, and *A. infectoria* cultures at final concentration of 10^4^ spores/g and incubated for 3 days. Non-inoculated sterilized grain samples used as control. DNA extracts isolated from the samples were analyzed using the qPCR assay. The obtained Ct values (23–30) revealed high reproducibility and demonstrated the ability to quantify fungal DNA in artificially contaminated wheat grains. qPCR analysis indicated the average values of DNA content for *F. proliferatum*, *A. flavus*, and *A. infectoria* spp. in seeds at concentrations of 268, 44 and 30 ng/mg, respectively.

### Mycotoxin Analysis

Detection, identification, and quantification of mycotoxins in wheat grain samples were performed using high-performance liquid chromatography coupled with tandem mass-spectrometry (LC/MS/MS) as described previously (Sadhasivam et al., 2017). Briefly, individual stock standard solutions (1 mg/ml) of aflatoxin B_1_, B_2_, G_1_, G_2_ (AFB_1_, AFB_2_, AFG_1_, AFG_2_), ochratoxin A (OTA), zearalenone (ZEN), deoxynivalenol (DON), fumonisin B_1_, B_2_ (FB_1_, FB_2_) and T-2 toxin (T-2) (Fermentek, Israel) were prepared in methanol. Multi-toxin working standard solutions with a series of toxin concentrations were prepared by dilution of the stock solutions of the analytes in methanol. For sample preparation, 5 g of the ground seeds were mixed with 20 ml of 25:75 (v/v) water/methanol and placed in an orbital shaker at 200 rpm for 30 min at room temperature. After centrifugation at 8500 ×*g* for 15 min, 5 ml of the supernatant were transferred to a 15-ml glass tube and evaporated under a stream of nitrogen at 50°C. The dry residue was reconstituted with 0.25 ml of a 95:5 (v/v) water/methanol mixture and centrifuged for 10 min at 17,000 ×*g*, at 4°C; the supernatant was used directly for the analysis. Three non-contaminated wheat grain samples were spiked with multi-mycotoxin standard solutions at three concentration levels for construction of calibration curves, which were used for mycotoxin quantification. The spiking experiments were performed in triplicate at three different time points. Chromatographic separation was carried out using Nexera X2 UHPLC (Shimadzu, Tokyo, Japan) equipped with 100 × 2.1 mm, 2.6 μm Kinetex C_18_ column, (Phenomenex, Torrance, CA, United States). The mobile phase consisted of 2.5 mM ammonium acetate acidified with 0.1% acetic acid (A), and methanol (B). The LC system was coupled with API 6500 hybrid triple quadrupole/linear ion trap mass spectrometer (Sciex, Concord, ON, Canada), equipped with a turbo-ion electrospray (ESI) ion source. Validation parameters, such as precision, accuracy, limit of detection (LOD), limit of quantification (LOQ), and specificity, were determined (Supplementary Table S3).

## Results

### Amplicon Sequencing

After paired-end alignments, quality filtering, and deletion of chimeric, singletons, and plant sequences, 22,338 16S rRNA gene sequences were assigned to 806 bacterial OTUs and 5,383,776 ITS sequences were assigned to 6,064 fungal OTUs. The number of 16S sequences varied between 186 and 5019 (Std. dev. 702) and ITS sequences varied between 23,948 and 359,517 (Std. dev. 42565) reads per sample. The number of OTUs, after rarefaction collapsing biological replicates, varied between 66 and 187 OTUs in the 16S rRNA gene dataset and between 350 and 515 OTUs in the ITS rRNA gene region dataset per category of samples (Supplementary Table S4).

### Wheat Grain Microbial Communities

**FIGURE 1:**
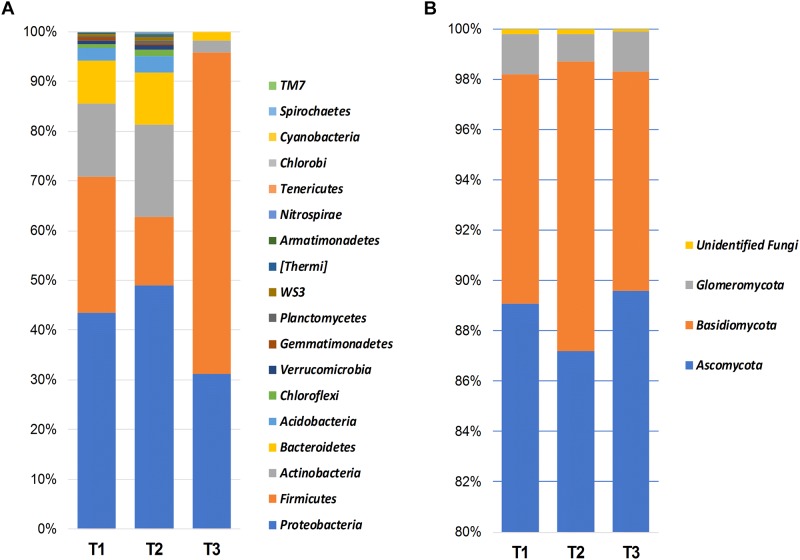
Bar charts showing the relative abundance of most dominant bacterial **(A)** and fungal **(B)** phyla detected using high-throughput sequencing technology across the samples collected at three storage time-points (T1, T2, T3).

Bacterial OTUs were mainly assigned to *Proteobacteria* (41.1%), *Firmicutes* (35.4%), *Actinobacteria* (11.9%), *Bacteroidetes* (6.9%), and *Acidobacteria* (1.9%) (Figure 1A). *Proteobacteria* was dominated by the presence of *Gammaproteobacteria* (21.7%), *Alphaproteobacteria* (8.2%), *Betaproteobacteria* (7.7%), and *Deltaproteobacteria* (3.3%).

Fungal members of the *Ascomycota* were the dominant fungal phylum across all samples, accounting for 88.5% of the total number of detected sequences (Figure 1B). This was followed by *Basidiomycota* (9.8%), *Glomeromycota* (1.4%), and unidentified fungi (0.2%). *Chytridiomycota*, *Entorrhizomycota*, *Mortierellomycota*, *Mucoromycota*, and *Olpidiomycota* were detected at a very low frequency (<0.1%). Ascomycota were largely identified as members of the classes *Dothideomycetes* (87.0%), and *Sordariomycetes* (1.1%). Whereas *Basidiomycota* were represented by *Tremellomycetes* (4.4%), *Microbotryomycetes* (2.8%), and *Pucciniomycetes* (1.5%), *Glomeromycota* was almost exclusively dominated by *Glomeromycetes* (1.4%).

### Compositional Differences in the Microbial Community After Different Lengths of Storage

**FIGURE 2:**
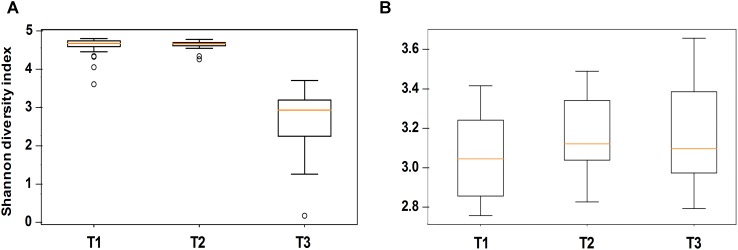
Alpha diversity analysis (based on Shannon diversity index) of the bacterial **(A)** and fungal **(B)** communities in stored wheat grain samples. Analyzed samples were collected immediately after harvest during the first week of storage (T1), after 3 months of storage (T2), and after 6 months of storage (T3). The centered square represents the mean, orange line inside the box represents the median, and circles indicate outliers.

**FIGURE 3:**
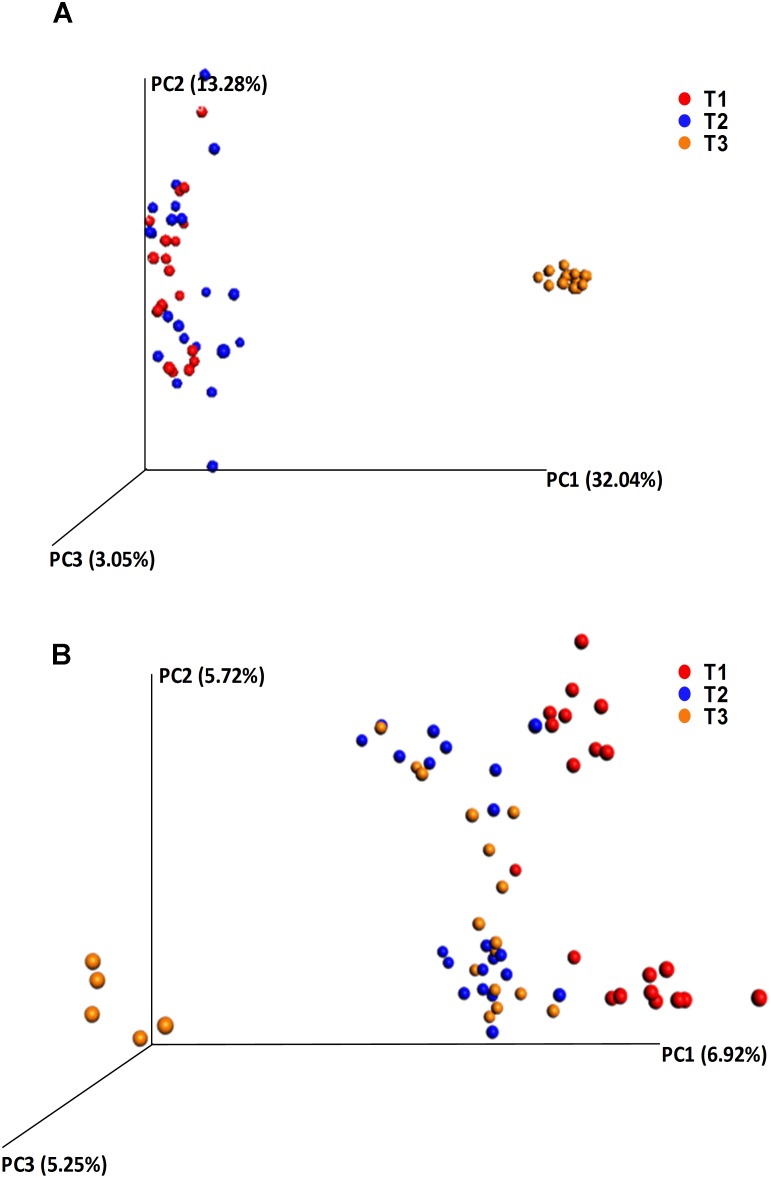
Principle coordinate analysis (PCoA) based on the beta diversity Bray Curtis dissimilarity metrics, showing the distance in the bacterial **(A)** and fungal **(B)** communities between the samples collected at T1 (red dots), T2 (blue dots), and T3 (orange dots).

Alpha diversity comparisons based on Shannon index, indicated that bacterial diversity varied significantly (*P* < 0.003) between T1 and T3, and T2 and T3 in both cases (Table 1 and Figure 2A). In contrast, Shannon index indicated no significant difference (*P* ≥ 0.1) in fungal community composition between the sampled time-points (Table 1 and Figure 2B). On the other hand, based on the Bray Curtis dissimilarity metric, the three time points (T1, T2, and T3) varied significantly (*P* < 0.01) in their bacterial and fungal community composition and structure (Table 1). The differences between the time points were also evident in the Principal Coordinate Analysis (PCoA) of the bacterial community, where samples from T3 were positioned far from T1 and T2 samples which formed a separate cluster (Figure 3A). PCoA analysis of the fungal community, however, did not provide any clear clustering based on sampling time (Figure 3B).

**Table 1:** *P-*values of the comparisons between the samples collected at three storage time-points (T1, T2, T3)^a^.

Microbial community	Comparison	Alpha diversity (Shannon index)^b^	Alpha diversity (Observed OTUs)^b^	Beta diversity (Bray Curtis)^c^
Bacteria	T1 vs. T2	0.228	0.174	0.010
	T2 vs. T3	0.003	0.003	0.001
	T1 vs. T3	0.003	0.003	0.001
Fungi	T1 vs. T2	0.222	0.003	0.001
	T2 vs. T3	0.1	0.429	0.003
	T1 vs. T3	0.624	0.003	0.001


*^a^*P-*values were determined using alpha diversity (observed OTUs and Shannon index) and beta diversity based on Bray Curtis metric. ^*b*^Index and statistical analysis conducted on a rarefied OTU table at an even sequencing depth. ^*c*^Index and statistical analysis conducted on a normalized OTU table using a CSS method.*

**FIGURE 4:**
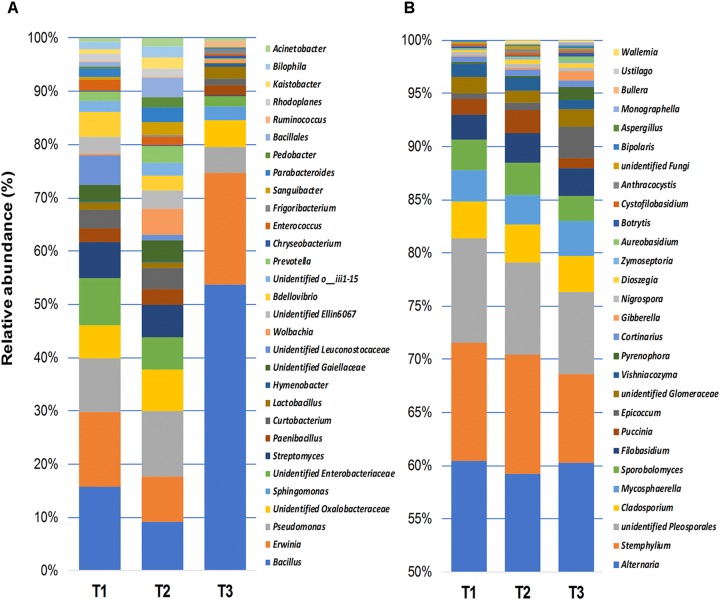
Relative abundance of bacterial (≥0.5%) **(A)**, and fungal (≥0.1%) **(B)** genera detected using high-throughput sequencing technology across the samples collected at three storage time-points (T1, T2, T3).

The relative abundance of fungal and bacterial genera detected in wheat grain samples across three sampling time points is shown in Figure 4. Bacterial communities of stored grains mainly consisted of genera of *Bacillus* (22.5%), followed by *Erwinia* (9.9%), *Pseudomonas* (5.9%), unidentified genera that belong to the family *Oxalobacteraceae* (4.3%), *Enterobacteriaceae* (3.7%), *Streptomyces* (3.3%), *Curtobacterium* (2.2%), and *Paenibacillus* (1.6%) (Figure 4A). Interestingly, *Bacillus* and *Erwinia* were the most abundant bacterial genera found at the last sampling time point (T3), which represented more than 70% of the total bacteria detected after phosphine fumigation (Figure 4A). In addition to the variations in community structure, the relative abundance of several other bacterial taxa, varied significantly between the different time points. Bacterial phylotypes that belongs to *Sphingomonas*, *Frigoribacterium*, *Ruminococcus*, *Hymenobacter*, *Acinetobacter, Chryseobacterium, Methylobacterium, Saccharibacillus, Staphylococcus, Agrobacterium*, *Pedobacter*, and *Comamonadaceae* were only present at the third sampling time point. In contrast, bacterial taxa such as *Ellin6067*, *Bdellovibrio*, *Prevotella*, *Bilophila*, *Parabacteroides*, and an unidentified group of *Leuconostocaceae* were present at almost equal relative abundance in T1 and T2 and then disappeared entirely in T3 (Supplementary Figure S2). Unlike bacteria, the most abundant fungal genera, such as *Alternaria* (59.2%), *Stemphylium* (10%), unidentified *Pleosporales* (8.6%), *Cladosporium* (3.4%), *Mycosphaerella* (3%), *Sporobolomyces* (2.7%), *Filobasidium* (2.6%), and *Puccinia* (1.5%), which accounted for more than 90% of the total fungal community, were equally present in all three of the sampled points (Figure 4B). Only three fungal genera, *Cortinarius*, *Dioszegia*, and *Monographella*, significantly changed in abundance over the three time points (Supplementary Figure S3).

### Isolation and Identification of Bacteria and Fungi From Wheat Grains

**FIGURE 5:**
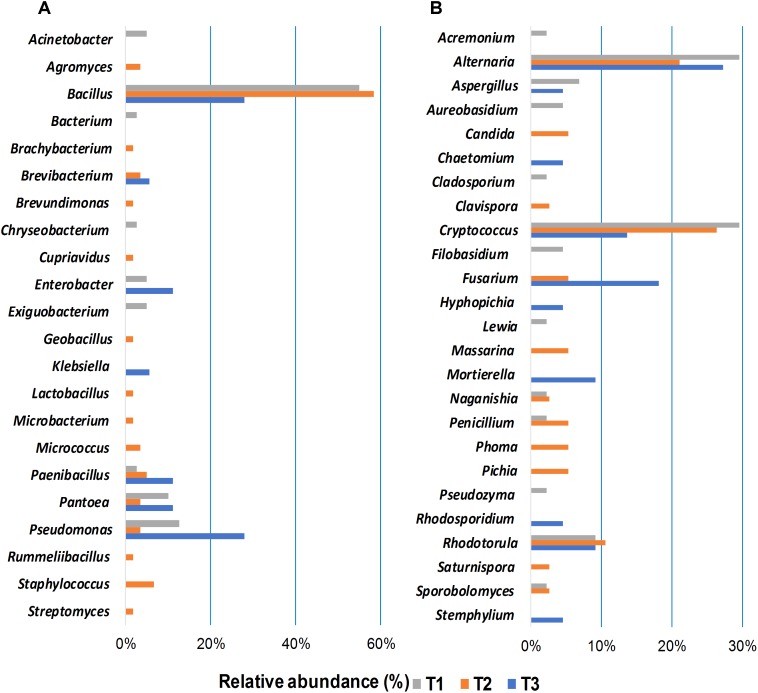
Relative abundance of bacterial **(A)**, and fungal **(B)** genera isolated by plating from grain samples collected at three storage time-points (T1, T2, T3). The isolates were identified by sequencing the 16S rRNA gene in bacteria and ITS region in fungi; the sequences were determined via BLAST matches to the NCBI database.

Bacterial and fungal isolates were cultured from wheat grains sampled at the three storage time points to assess the impact of phosphine fumigation on microbial diversity and to evaluate potential interactions between the members of the shared microbiome. Forty bacterial and 44 fungal (24 yeasts and 20 filamentous fungi) isolates were cultured from seeds at the first time point (Figure 5). *Bacillus* spp. were predominant among cultured bacterial community (55%) followed by *Pseudomonas* spp. (12.5%) and *Pantoea* spp. (10%) (Figure 5A). *Cryptococcus* and *Rhodotorula* species predominated among the isolated yeasts cultures (39%) based on morphological features (colony color, texture, microscopic observation) and sequencing analysis. The same criteria indicated that 13 out of 44 cultured filamentous fungal isolates were *Alternaria* spp. (30%), followed by *Aspergillus* spp. (7%) (Figure 5B).

A more diverse bacterial community was identified in the bacterial isolates cultured at the second time point (60 isolates), where *Bacillus* spp. were most common (58%) (Figure 5A). The 38 fungal isolates cultured from wheat grains at the second time point were identified as *Alternaria* spp. (21%), *Fusarium* spp., *Massarina* spp., *Penicillium* spp., and *Phoma* spp. (5% for each genus). *Cryptococcus* and *Rhodotorula* spp. were still the most abundant (36%) yeast taxa (Figure 5B). A dramatic decrease was observed in the number of bacterial strains isolated from wheat grains sampled at the third time point (18 isolates). *Bacillus* and *Pseudomonas* spp. were the predominant isolates cultured from T3 samples (28% for each genus) (Figure 5A). The number of yeast isolates cultured from T3 samples was also significantly reduced compared to T1 and T2 (Figure 5B). Fungal abundance and diversity in T3 samples did not change, however, relative to T1 and T2. Fifteen filamentous fungal isolates, out of a total 22 fungal microorganisms, were cultured from stored seeds collected at the T3 time point. Mycotoxigenic fungi, such as *Alternaria* (27%) and *Fusarium* (18%) species, were predominant. The changes in microbial community composition identified in the culturing assay were in good agreement with results obtained in the alpha- and beta-diversity analyses (Figure 2, 3).

### Analysis of Mycotoxigenic Fungi in Stored Grains by Real-Time PCR

Since cultured fungal diversity was not influenced by phosphine fumigation and was generally consistent across the three sampled time points, real-time PCR method was utilized to quantify fungal DNA in stored grain samples. The broad-spectrum primers and specific fluorescent probes were used to amplify the targeted genomic DNA and to detect and quantify the major mycotoxigenic fungal genera, *Aspergillus*, *Fusarium*, and *Alternaria*.

**FIGURE 6:**
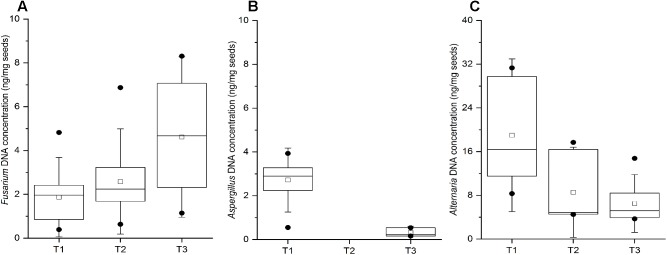
Quantification by qPCR of *Fusarium* spp. **(A)** *Aspergillus* spp. **(B)**, and *Alternaria* spp. **(C)** in stored wheat grain at three storage time-points (T1, T2, T3). qPCR results are from at least two biological replicates (DNA extractions) and three technical replicates per sample. The lower and upper edges of each box correspond to the 25th and 75th percentiles. The black line inside the box represents the median, and centered square represents the mean. The whiskers represent 1.5× interquartile range. Dots indicate outliers.

The wheat grain samples collected throughout the entire study did not exhibit any evidence of spoilage. Nevertheless, the qPCR analysis indicated the presence of all three pathogenic genera in majority of the samples, which corroborates the results obtained in the culture assay of wheat grains. *Fusarium* DNA was detected in 11, 12, and 15 out of 21 wheat grain samples at T1, T2, and T3 time points, respectively, with an increased DNA content at T3 (Figure 6A). The obtained Ct values ranging between 27.62 and 32.78 indicated the presence of relatively low concentrations of *Fusarium* DNA in the samples (0.5 – 8 ng/mg; Figure 6A), suggesting that qPCR is more sensitive in detecting mycotoxigenic fungi compared to culture-based approaches. *Aspergillus* DNA was detected in only 10 and 3 out of 21 wheat grain samples collected at the T1 and T3 time points, respectively, with no detection in the T2 samples (Figure 6B). In contrast, 9 to 11 wheat grain samples in all three time points were found to be positive for *Alternaria* spp. by qPCR, and with a relatively higher fungal DNA content relative to the other tested mycotoxigenic species (Figure 6C).

### Mycotoxin Detection in Stored Wheat Grains

The LC/MS/MS multi-toxin method was optimized for the simultaneous detection and quantification of 10 mycotoxins in wheat grain samples to investigate potential changes in mycotoxin levels in grains over the storage period of 6 months, including after phosphine fumigation for insect control. Results indicated that 17 wheat samples were positive for mycotoxin contamination and were above their limit of detection (LOD) but under the maximum EU regulatory limits (Table 2). AFB_2_ was detected at 0.4 ppb in three wheat samples collected at T1 and the co-occurrence of AFG_2_ was only observed in one of the samples. Two grain samples from T3 were also found to be contaminated with AFB_2_ (Table 2). These findings are consistent with data obtained in the culturing assay and by qPCR, where *Aspergillus* spp. were also detected in T1 and T3 samples. The LC/MS/MS analysis also indicated the presence of fumonisins at values below the regulatory guidelines in the samples analyzed at all three time points. Notably, a strong link was observed between increased abundance of *Fusarium* spp. in T3 wheat grain samples, and the presence of DON toxin only in T3 samples (Table 2). These results indicate that phosphine fumigation significantly altered the structure of microbial community of stored wheat grains and induced shift in the abundance of mycotoxin-producing *Fusarium* taxa.

**Table 2:** Mycotoxin contamination in stored wheat grain samples (ng/g)^a,b^.

Sampling time-points	Sample ^#^	AFB_1_	AFB_2_	AFG_1_	AFG_2_	FB_1_	FB_2_	DON	OTA	T-2	ZEN
T1	1	–	–	–	–	2.3 ± 0.48	–	–	–	–	–
	8	–	–	–	–	1.7 ± 0.23	–	–	–	–	–
	9	–	0.4 ± 0.01	–	–	–	–	–	–	–	–
	11	–	–	–	–	–	9.4 ± 1.75	–	–	–	–
	14	–	–	–	–	1.9 ± 0.09	–	–	–	–	–
	16	–	0.4 ± 0.012	–	–	2.0 ± 0.14	–	–	–	–	–
	17	–	0.4 ± 0.002	–	1.9 ± 0.01	3.4 ± 0.12	–	–	–	–	–
T2	22	–	–	–	–	2.4 ± 0.02	–	–	–	–	–
	24	–	–	–	–	–	9.5 ± 0.78	–	–	–	–
	25	–	–	–	–	–	–	–	–	–	–
	30	–	–	–	–	2.5 ± 0.02	–	–	–	–	–
T3	45	–	0.4 ± 0.017	–	–	2 ± 0.01	–	–	–	–	–
	46	–	–	–	–	–	–	38.8 ± 1.90	–	–	–
	47	–	0.4 ± 0.022	–	–	–	–	–	–	–	–
	48	–	–	–	–	–	–	21.2 ± 1.10	–	–	–
	51	–	–	–	–	1.4 ± 0.08	–	–	–	–	–
	58	–	–	–	–	–	–	144.1 ± 9.1	–	–	–
	60	–	–	–	–	–	–	7.7 ± 0.69	–	0.2 ± 0.001	–


*^a^The table represents the positive samples data only. ^*b*^Average values of mycotoxin concentration ± standard error (the measurements were repeated 3 times). “–” not detected.*

## Discussion

An objective of the present study was to characterize the composition of microbial communities associated with stored wheat grains and to identify any changes in the diversity and composition of the wheat microbiome during storage and in response to phosphine fumigation to control insect pests. Characterizing the microbiota of wheat grains after harvest is an essential step to understanding the interactions that potentially occur between different members of the community in relation to the colonization of the grains with common mycotoxigenic fungi (*Aspergillus*, *Fusarium*, *Penicillium*, and *Alternaria* species). Results of the HTS analysis indicated that fumigation of the grains with phosphine had a significant effect on the diversity and abundance of various components of the wheat-grain microbiome. It is known that many other factors could affect the stored seed microbial community such as temperature, humidity, water activity, grain moisture (Schmidt et al., 2018). Nevertheless, in the current study, the impact of such environmental factors was minor due to the optimal and stable weather conditions during a 6-months storage period. In general, the composition of the bacterial and fungal microbiota of wheat grains found in the current study is in agreement with previous studies (Karlsson et al., 2014, 2017; Comby et al., 2016; Díaz Herrera et al., 2016; Gdanetz and Trail, 2017). Genera, including *Bacillus, Erwinia*, *Pseudomonas*, and *Paenibacillus* were dominant bacterial taxa identified, while *Alternaria*, *Stemphylium*, *Cladosporium*, *Sporobolomyces*, *Mycosphaerella*, and *Filobasidium* were the most prevalent fungi and represent the most common members of the wheat grain mycobiome. The majority of the detected bacteria taxa, with the exception of *Erwinia*, are known to have a beneficial impact on plants. For instance, *Bacillus* species are known to have a growth promotive effect on wheat plants and are generally isolated from both grains and the rhizosphere (Tao et al., 2014; Pan et al., 2015; Cherif-Silini et al., 2016). *Paenibacillus* is recognized as a predominantly endophytic bacterium in wheat plants and seeds, also with growth promotive effects (Díaz Herrera et al., 2016; Hao and Chen, 2017). Similarly, *Pseudomonas* contains several species that promote plant growth by suppressing pathogenic microorganisms, and synthesizing growth-stimulating plant hormones (Preston, 2004). Notably, however, other species of *Pseudomonas* may be phytopathogenic. In the present study, the results obtained with the culturing assay of stored wheat grains microbes were in agreement with the results obtained by HTS sequencing data. *Bacillus*, *Pseudomonas*, and *Pantoea* were found to be most abundant genera obtained in the cultured bacterial taxa isolated from stored wheat grains throughout the study, and the same was true in the HTS analysis.

The data indicated that phosphine fumigation significantly affected the microbial community associated with wheat grains. Phosphine or hydrogen phosphide (PH_3_) is a low molecular weight compound that diffuses rapidly and penetrates deeply into materials, such as the bulk storage of grain (Bond, 1989). It is the dominant fumigant used to control insect pests in stored grain and many other stored commodities. Since phosphine is toxic to aerobically respiring organisms, it also has effects on the survival and growth of some aerobic bacteria and fungi (Hocking and Banks, 1991; Castro et al., 2000). In the present study, bacterial population exhibited a drastic reduction in their level of diversity and the number of observed species after fumigation. Highly abundant bacteria, including *Streptomyces*, *Ellin606*, *Bdellovibrio*, *Prevotella*, *Bilophila*, and unidentified groups of *Parabacteroides*, and *Leuconostocaceae* that collectively accounted for approximately 45% of the total bacterial OTUs in the first two time points (T1 and T2), disappeared entirely in T3 after the phosphine treatment. This change corresponded with a significant increase in *Bacillus* from an average of 2.2% in T1 and T2 to more than 50% in T3 (Supplementary Figure S2). A similar trend was reported by Yuan et al. (2018), who reported that the abundance of *Bacillus* spp. in wheat grains increased from 2 to 36% after 9 months of storage and up to 48% after 12 months of storage at different position within a storage silo. Interestingly, in that study, the authors indicated that stored wheat grain was treated with ozone to kill insects.

Small quantities of stable but harmless breakdown products of phosphine, which become incorporated into normal cellular metabolism as phosphates and phosphites, may remain in fumigated materials (Nath et al., 2011). *Bacillus* and *Pseudomonas* spp. are capable of utilizing phosphite and belong to the group of phosphate solubilizing bacteria, which play an important role in plant growth promotion (Foster et al., 1978; Metcalf and Wolfe, 1998; Igual et al., 2001; Park et al., 2009; Kang et al., 2014). *Bacillus* and *Pseudomonas* may take advantage of these unique abilities after phosphine fumigation to enhance their survival and proliferation. The exclusive increase in the abundance of *Sphingomonas* species, which are aerobic bacteria utilized for environmental remediation due to their ability to degrade aromatic compounds (Story et al., 2004), after phosphine fumigation, may indicate their involvement in the degradation of this toxic substance. Since one of the main modes of action of phosphine is the inhibition of *cytochrome c oxidase* (Price, 1980), apparently some oxidase-negative bacteria (*i.e.*, those that do not rely on cytochrome c oxidase in respiration), such as *Acinetobacter*, *Lactobacillus, Ruminococcus, Methylobacterium, Saccharibacillus*, and *Frigoribacterium* species, may survive and proliferate following phosphine treatment.

Results indicated that neither the diversity nor abundance of fungi were affected by phosphine fumigation. Only a few studies have described the influence of phosphine on the survival and growth of molds in stored grains. Phosphine was reported to have little effect on populations of fungi that were unable to grow in stored grains under conditions of limiting water activity (a_w_ 0.8–0.86) (Raghunathan et al., 1969; Sinha et al., 1967; Hocking and Banks, 1991). Natarajan and Bagyaraj (1984) noted some reduction in fungal growth on legumes when exposed to very high phosphine levels (100 g/m^3^), particularly at limiting a_w_ conditions (0.8). It seems that the effect of phosphine on fungal growth, however, may vary between fungal species and the type of stored grain or seed. Phosphine was shown to inhibit the growth of *A. flavus* and/or *A. parasiticus* and aflatoxin production on peanuts in both laboratory and warehouse experiments (Castro et al., 1992, 1995, 1996). A reduction of *A. flavus* growth and aflatoxin production was also observed in response to phosphine treatment of maize kernels stored at different moisture levels (Castro et al., 2000). In the same study, however, the authors stated that *Penicillium* species and *F. verticillioides*, which are commonly found in freshly harvested grains, were tolerant to phosphine (Castro et al., 2000). Those findings are consistent with the results obtained in the current study of wheat-grain-associated microflora, in which a greater number of *Fusarium* species, such as *F. culmorum* and *F. proliferatum*, were detected at the last sampling time point (T3), probably due to the changes that occurred in microbial community composition following phosphine fumigation. Notably, the presence of mycotoxigenic *Fusarium* isolates in the stored wheat grains after fumigation was strongly associated with the occurrence of DON in T3 samples, suggesting that phosphine induced a shift in the microbial composition toward more toxigenic strains. Shifts in *Fusarium* species composition on cereal grain due to changes in climate and cultivation practices have been previously reported (Garrett et al., 2011). Magan et al. (2011) investigated the effect of climate change on mycotoxin-producing fungi and indicated that shifts toward *Fusarium* pathogens and DON contamination in cereals may occur due to changes in management practices. For example, the effect of fungicides on *Fusarium* infections could be different depending on the antifungal compound, the time of treatment, and the composition of the microbial flora in cereals (Pirgozliev et al., 2003). Henriksen and Elen (2005) reported that different fungicides (such as propiconazole and cyprodinil) significantly increased the infection level of DON-producing *F. avenaceum*, *F. tricinctum*, and *F. culmorum* in grains.

## Conclusion

In summary, our study demonstrated that phosphine fumigation had a significant impact on microbial community composition in stored wheat grain. The shifts in bacterial and yeast populations, coincident with the fumigant application may have also led to changes in the functional diversity of those communities. Fumigation did not change filamentous fungal abundance in stored grains at T3; however, phosphine treatment apparently altered the community composition of molds, relative to T1 and T2. Previous studies have extensively investigated the effect of different fumigants on soil bacterial communities (Engelen et al., 1998; Ibekwe et al., 2001; van Agtmaal et al., 2015; Zhang et al., 2017). To our knowledge, however, there are no published studies describing the impact of pesticide treatment on the microbiome of stored wheat grains using combined HTS analysis and culture assays. The development of next generation sequencing (NGS) technologies have provided a deeper understanding of the microbiome on plant material, as well human and animal subjects, and a host of different physical environments. Since many of the microorganisms identified by NGS technology are not culturable, it would be very difficult to detect alterations in microbial community diversity and abundance after phosphine treatment using only classical culturing and molecular identification approaches. Findings in the current study are consistent with previous studies, where NGS technology was able to identify changes in the microbiome that were not detectable using classical methodologies (Sylla et al., 2013; Schmidt et al., 2014). A better understanding of the interactions that occur among the different members of the microbial community of stored wheat grains at specific time points (e.g., before and/or after fumigant application) can assist in the prediction of fungal disease incidence and mycotoxin production, as well as in the development of novel approaches for controlling mycotoxin contamination in grains and managing crop diseases in general.

## Data Availability

The raw sequence files supporting the findings of this article are available in the NCBI Sequence Read Archive (SRA) under the BioProject ID PRJNA503713.

## Author Contributions

MKS, AA, SD, and ES conceived and designed the experiments, analyzed the data, and wrote the manuscript. MKS, AA, MB, VZ, and MW performed the experiments. MW provided critical comments on the study and edited the manuscript. All authors read and approved the final manuscript.

## Conflict of Interest Statement

The authors declare that the research was conducted in the absence of any commercial or financial relationships that could be construed as a potential conflict of interest.

## Footnotes

1 www.illumina.com

2 https://blast.ncbi.nlm.nih.gov/Blast.cgi
