# Supplementary material for: Shifts in the Composition of the Microbiota of Stored Wheat Grains in Response to Fumigation
Source: Front Microbiol. 2019 May 17;10:1098. doi: 10.3389/fmicb.2019.01098 (PMC6533538; doi:10.3389/fmicb.2019.01098)
Supplement: Supplementary file 1 [file Table_1.DOCX]

Supplementary Material

Figures and tables

# Supplementary tables

**Table S1. List of primers used in the study**

| Target | Primer name | Sequence (5′-3′) | Reference | PCR conditions |
| --- | --- | --- | --- | --- |
| ^a^16S rRNA | 515F | *ACACTGACGACATGGTTCTACA*GTGCCAGCMGCCGCGGTAA | (Caporaso et al., 2012) | 94°C-3 min; 35 cycles (94°C-45s, 50°C-60s & 72°C-90s); 72°C-10 min |
|  | 926R | *TACGGTAGCAGAGACTTGGTCT*CCGYCAATTYMTTTRAGTTT | (Quince et al., 2011) |  |
| ^a^ ITS | ITS 5F | *TCGTCGGCAGCGTCAGATGTGTATAAGAGACAG*GGAAGTAAAAGTCGTAACAAGG | (Scibetta et al., 2018) | 98°C-3 min; 30 cycles (95°C-30s, 50°C-30s & 72°C-30s); 72°C-1 min |
|  | ITS 86R | *GTCTCGTGGGCTCGGAGATGTGTATAAGAGACAG*TTCAAAGATTCGATGATTCAC |  |  |
| ^b^16S rRNA | 27F | AGAGTTTGATCMTGGCTCAG | (Weisburg et al., 1991) | 95°C-10 min, 35 cycles (90°C-30s, 50°C-30s & 72°C-2 min); 72°C-5 min |
|  | 1492R | TACGGYTACCTTGTTACGACTT |  |  |
| ^b^ ITS | ITS1 | TCCGTAGGTGAACCTGCGG | (Mitchell et al., 1994) | 95°C-3 min, 30 cycles (95°C-30s, 56°C-60s & 72°C-90s); 72°C-5 min |
|  | ITS4 | TCCTCCGCTTATTGATATGC |  |  |

^a^ Illumina miSeq sequencing (adapter sequences are italicized)

^b^ Sanger sequencing

**Table S2. Real-time primers and probes used in this study**

| Primers/probes | Sequence (5’-3’) | Reporter/quencher ^a^ | Reference | PCR conditions |
| --- | --- | --- | --- | --- |
| ITSPF | GGCATCGATGAAGAACGCAGC |  | (Suanthie et al., 2009) | 50°C-2 min; 95°C-2 min; 45 cycles (95°C-15s,55°C-15s & 60°C-15s) |
| ITSPR | CCTACCTGATCCGAGGTCAAC |  |  |  |
| ASP1P | CAAGCMCGGCTTGTGTGTTGGGTCGYCGTC | FAM/TAMRA |  |  |
| FUSP | ACGCTCGAACAGGCATGCCCGCCAGAATAC | TEX/BHQ2 |  |  |
| AltTM | AACACCAAGCAAAGCTTGAGGGTACAAAT | HEX/BHQ1 | (Pavón et al., 2012) |  |

^a^ Probes were labeled at the end 5’-ends with the reporter (FAM, TEX, and HEX) and at the 3’-ends with quenchers (TAMRA, BHQ1, and BHQ2).

**References**

Caporaso, J. G., Lauber, C. L., Walters, W. A., Berg-Lyons, D., Huntley, J., Fierer, N., et al. (2012). Ultra-high-throughput microbial community analysis on the Illumina HiSeq and MiSeq platforms. *ISME J.* 6, 1621–1624. doi:10.1038/ismej.2012.8.

Mitchell, T. G., Freedman, E. Z., White, T. J., and Taylor3, J. W. (1994). Unique oligonucleotide primers in PCR for identification of *Cryptococcus neoformans*. *J. Clin. Microbiol.*, 253–255. Available at: http://jcm.asm.org/content/32/1/253.full.pdf [Accessed April 16, 2018].

Pavón, M. Á., González, I., Martín, R., and García Lacarra, T. (2012). ITS-based detection and quantification of Alternaria spp. in raw and processed vegetables by real-time quantitative PCR. *Food Microbiol.* 32, 165–171. doi:10.1016/j.fm.2012.05.006.

Quince, C., Lanzen, A., Davenport, R. J., and Turnbaugh, P. J. (2011). Removing noise from pyrosequenced amplicons. *BMC Bioinformatics* 12, 38. doi:10.1186/1471-2105-12-38.

Scibetta, S., Schena, L., Abdelfattah, A., Pangallo, S., and Cacciola, S. O. (2018). Selection and Experimental Evaluation of Universal Primers to Study the Fungal Microbiome of Higher Plants. *Phytobiomes J.* 2, 225–236. doi:10.1094/PBIOMES-02-18-0009-R.

Suanthie, Y., Cousin, M. A., and Woloshuk, C. P. (2009). Multiplex real-time PCR for detection and quantification of mycotoxigenic *Aspergillus*, *Penicillium* and *Fusarium*. *J. Stored Prod. Res.* 45, 139–145. doi:10.1016/J.JSPR.2008.12.001.

Weisburg, W. G., Barns, S. M., Pelletier, D. A., and Lane, D. J. (1991). 16S ribosomal DNA amplification for phylogenetic study. *J. Bacteriol.* 173, 697–703. Available at: http://www.ncbi.nlm.nih.gov/pubmed/1987160 [Accessed April 16, 2018].

**Table S3. Summary of LC/MS/MS validation results**

| **Compound** | **Precursor Ion m/z** | **Quantifier (Qualifier) Ions m/z** | **Calibration Level (ppb)** | **Calculated Concentration ppb (±SD) ^a^** | **RSD (%) ^b^** | **LOQ (LOD) ppb** |
| --- | --- | --- | --- | --- | --- | --- |
| AFB_1_ | 313  [M + H]^+^ | 285 (128) | 1 | 0.99 (0.1) | 10.1 | 0.5  (0.1) |
|  |  |  | 2 | 1.98 (0.15) | 7.2 |  |
|  |  |  | 16 | 16.1 (0.38) | 2.6 |  |
| AFB_2_ | 315  [M + H]^+^ | 287 (259) | 1 | 1.09 (0.09) | 7.8 | 0.4  (0.1) |
|  |  |  | 2 | 2.05 (0.1) | 4.9 |  |
|  |  |  | 16 | 15.9 (0.81) | 5.3 |  |
| AFG_1_ | 329  [M + H]^+^ | 243 (200) | 1 | 1.06 (0.12) | 10.8 | 0.5  (0.2) |
|  |  |  | 2 | 2.03 (0.12) | 6.0 |  |
|  |  |  | 16 | 15.8 (1.01) | 6.8 |  |
| AFG_2_ | 331  [M + H]^+^ | 313 (245) | 1 | 1.08 (0.11) | 8.8 | 1.0  (0.3) |
|  |  |  | 2 | 2.1 (0.1) | 4.8 |  |
|  |  |  | 16 | 15.7 (0.4) | 2.9 |  |
| FB_1_ | 722  [M + H]^+^ | 334 (352) | 20 | 20.2 (0.88) | 6.1 | 8.6  (2.4) |
|  |  |  | 40 | 39.8 (1.4) | 4.2 |  |
|  |  |  | 320 | 326.4 (24.8) | 8.1 |  |
| FB_2_ | 706  [M + H]^+^ | 336 (318) | 20 | 21.7 (3.6) | 18.4 | 7.3  (2.4) |
|  |  |  | 40 | 39.8 (4.9) | 12.9 |  |
|  |  |  | 320 | 330.3 (20.7) | 7.7 |  |
| DON | 355  [M + CH_3_COO]^−^ | 295 (265) | 40 | 40.5 (3.5) | 7.9 | 11.0  (3.1) |
|  |  |  | 80 | 80.2 (4.6) | 6.2 |  |
|  |  |  | 640 | 637.5 (27.1) | 4.3 |  |
| OTA | 404  [M + H]^+^ | 239 (102) | 2 | 2.1 (0.2) | 9.9 | 0.5  (0.1) |
|  |  |  | 4 | 3.9 (0.24) | 5.1 |  |
|  |  |  | 32 | 32.2 (2.9) | 8.7 |  |
| T-2 | 484  [M + NH_4_]^+^ | 215 (185) | 40 | 44.7 (5.5) | 14.8 | 8.1  (2.4) |
|  |  |  | 80 | 83.9 (8.1) | 11.9 |  |
|  |  |  | 640 | 645.6 (15.8) | 4.2 |  |
| ZEN | 317  [M − H]^−^ | 131 (175) | 20 | 21.8 (4.9) | 18.5 | 6.2  (1.5) |
|  |  |  | 40 | 41.1 (2.1) | 6.1 |  |
|  |  |  | 320 | 327.8 (19.5) | 8.5 |  |

^a^ Calculated concentrations (±SD) for each calibration level represent the accuracy of measurements

^b^ The relative standard deviation (RSD) represents the repeatability

**Table S4. Number of bacterial and fungal OTUs detected in each sample after collapsing three biological replicates**

| Storage Time | Sample ID | Location | Bacterial OTUs ^a^ | Fungal OTUs ^a^ |
| --- | --- | --- | --- | --- |
| T1 | 0Mw1 | Warehouse #1 Emek Israel (Northern Israel) | 81 | 355 |
|  | 0Mw2 | Warehouse #2 Emek Israel (Northern Israel) | 119 | 350 |
|  | 0Mw3 | Warehouse #3 Emek Israel (Northern Israel) | 66 | 381 |
|  | 0Nw1 | Warehouse #1 Netivot (Southern Israel) | 114 | 380 |
|  | 0Yw1 | Warehouse #1 Yeshiviya (Southern Israel) | 92 | 413 |
|  | 0Yw2 | Warehouse #2 Yeshiviya (Southern Israel) | 93 | 413 |
|  | 0Yw3 | Warehouse #3 Yeshiviya (Southern Israel) | 143 | 412 |
| T2 | 3Mw1 | Warehouse #1 Emek Israel (Northern Israel) | 104 | 515 |
|  | 3Mw2 | Warehouse #2 Emek Israel (Northern Israel) | 76 | 463 |
|  | 3Mw3 | Warehouse #3 Emek Israel (Northern Israel) | 110 | 462 |
|  | 3Nw1 | Warehouse #1 Netivot (Southern Israel) | 75 | 491 |
|  | 3Yw1 | Warehouse #1 Yeshiviya (Southern Israel) | 72 | 505 |
|  | 3Yw2 | Warehouse #2 Yeshiviya (Southern Israel) | 105 | 457 |
|  | 3Yw3 | Warehouse #3 Yeshiviya (Southern Israel) | 104 | 467 |
| T3 | 6Mw1 | Warehouse #1 Emek Israel (Northern Israel) | 122 | 402 |
|  | 6Mw2 | Warehouse #2 Emek Israel (Northern Israel) | 187 | 478 |
|  | 6Mw3 | Warehouse #3 Emek Israel (Northern Israel) | 138 | 447 |
|  | 6Nw1 | Warehouse #1 Netivot (Southern Israel) | 126 | 472 |
|  | 6Yw1 | Warehouse #1 Yeshiviya (Southern Israel) | 142 | 491 |
|  | 6Yw2 | Warehouse #2 Yeshiviya (Southern Israel) | 182 | 469 |
|  | 6Yw3 | Warehouse #3 Yeshiviya (Southern Israel) | 150 | 464 |

^a^ The number OTUs was calculated an even sampling depth

# Supplementary figures
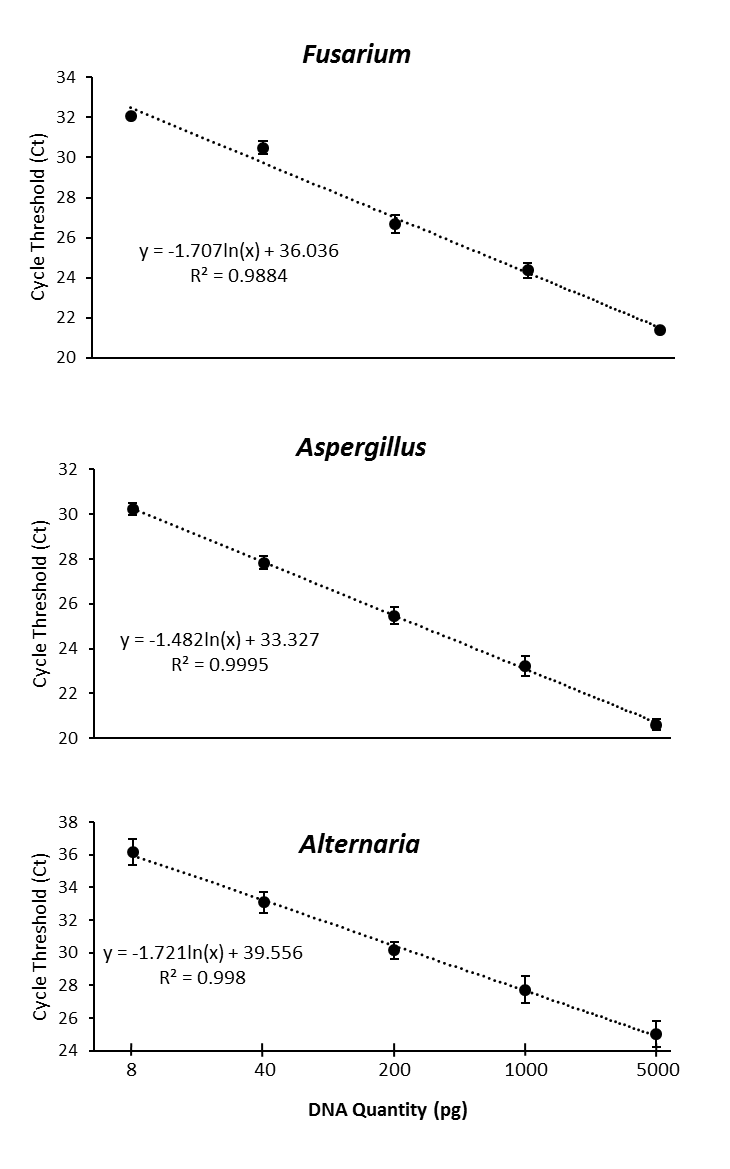


**FIGURE S1:** Standard curves generated for *Fusarium*, *Aspergillus*, and *Alternaria*. Genomic DNAs from *F. proliferatum*, *A. flavus* and *A. infectoria* were serially diluted 5-fold and analyzed by the genus-specific real-time PCR. The initial DNA concentration was 5 ng/µl. Cycle thresholds (Ct) were plotted against the DNA concentrations. Error bars indicate the standard deviations of Ct among 3 replicates.





**FIGURE S2:** Bacterial taxa detected with a significantly different relative abundance in grain samples and their distribution between three storage time-points (T1, T2 and T3). The comparison of the most abundant genera was determined using Kruskal–Wallis test





**FIGURE S3:** Fungal taxa detected with a significantly different relative abundance in grain samples and their distribution between three storage time-points (T1, T2 and T3). The comparison of the most abundant genera was determined using Kruskal–Wallis test
